# Supplementary material for: Genomic epidemiology of SARS-CoV-2 under an elimination strategy in Hong Kong
Source: Nat Commun. 2022 Feb 8;13:736. doi: 10.1038/s41467-022-28420-7 (PMC8825829; doi:10.1038/s41467-022-28420-7)
Supplement: Supplementary file 1 — Supplementary Information [file 41467_2022_28420_MOESM1_ESM.pdf]

# Supplementary Information

# Genomic epidemiology of SARS-CoV-2 under an elimination strategy in Hong Kong

Haogao Gu[1,6], Ruopeng Xie[1,2,6], Dillon C. Adam[1,6], Joseph L.-H. Tsui[1,6], Daniel K. Chu[1,6], Lydia D.J Chang[1], Sammi S.Y. Cheuk[1], Shreya Gurung[1], Pavithra Krishnan[1], Daisy Y.M. Ng[1], Gigi Y.Z. Liu[1], Carrie K.C. Wan[1], Samuel S.M. Cheng[1], Kimberly M. Edwards[1,2], Kathy S.M. Leung[1,3], Joseph T. Wu[1,3], Dominic N.C. Tsang[4], Gabriel M. Leung[1,3], Benjamin J. Cowling[1,3], Malik Peiris[1,2,5], Tommy T.Y. Lam[1,3,5], Vijaykrishna Dhanasekaran[1,2,*], Leo L.M. Poon[1,2,5,*]

[1]School of Public Health, LKS Faculty of Medicine, The University of Hong Kong, Hong Kong, China.

[2]HKU-Pasteur Research Pole, School of Public Health, LKS Faculty of Medicine, The University of Hong Kong, Hong Kong, China.

[3]Laboratory of Data Discovery for Health, Hong Kong Science and Technology Park, Hong Kong, China.

[4]Centre for Health Protection, Department of Health, The Government of Hong Kong Special Administrative Region, Hong Kong, China.

[5]Centre for Immunology & Infection, Hong Kong Science and Technology Park, Hong Kong, China.

[6]These authors contributed equally

*To whom correspondence should be addressed. Email: veej@hku.hk, llmpoon@hku.hk

**Supplementary Note 1: Deep sequencing summary statistics**

SARS-Cov-2 sequences from Hong Kong contained within-patient variation in 12,859 sites of the genome when compared to the SARS-CoV-2 reference strain Wuhan-Hu-1 (GenBank: MN908947.3). 37.2% of the sites (n=4,779) contained mutations in more than one sample. High frequency of variation (in >100 Hong Kong sequences) was observed in 30 sites (Supplementary Table 6). The spectrum of allele frequencies (Supplementary Fig. 8) showed that over 90% of the variants had allele frequency ≥95% or ≤10%. We observed two variants unique to Hong Kong, C5812T and G25785T, which were detected in separate phylogenetic clusters of local Hong Kong cases. The C5812T mutation was identified in two sperate clusters in the fourth wave. While the C5812T mutation in one cluster likely descended from a local ancestral case, the mutation in the earlier cluster may have been imported. Similarly, G25785T was found in both the third and fourth waves, and mutations in at least one cluster likely originated from local cases. Some of the low-frequency SNVs (allele frequency <5%, shown in the low peaks to the bottom of Supplementary Fig. 8 and Supplementary Table 7) commonly occurred in global context. For example, the G28883C (G205R in nucleocapsid) and C22227T (A665V in spike) mutations were found in 38.09% and 21.49% of the global cases, however they were only seen in 1.56% and 0.7% of the Hong Kong cases respectively.

## Supplementary Figures

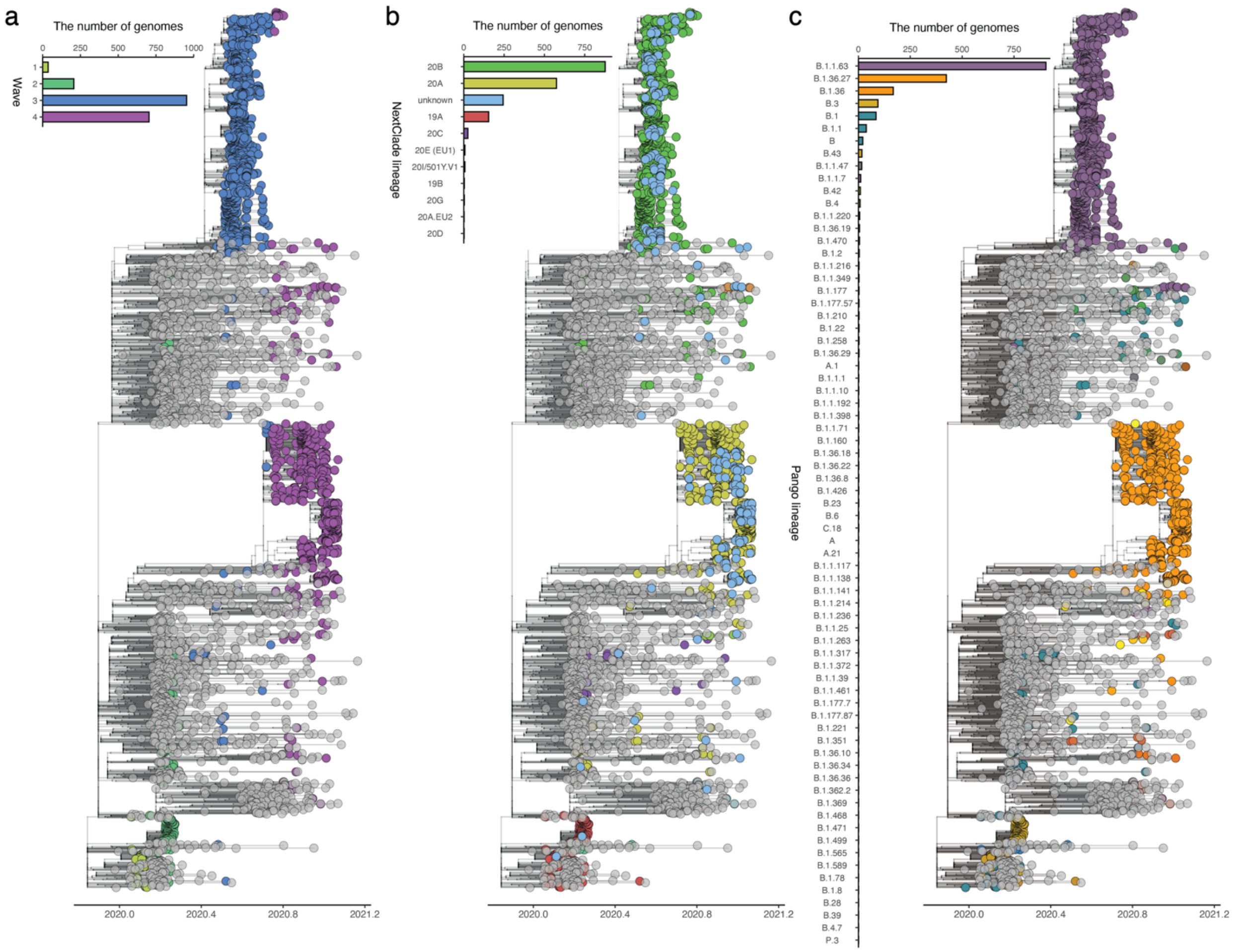


**Supplementary Fig. 1 | Time-scaled maximum-likelihood phylogeny of SARS-CoV2 using IQ-TREE (v.2).** The tree colored by (**a**) pandemic waves in Hong Kong, (**b**) Nextclade, and (**c**) PANGO classification, respectively. Global sequences are shown in grey.

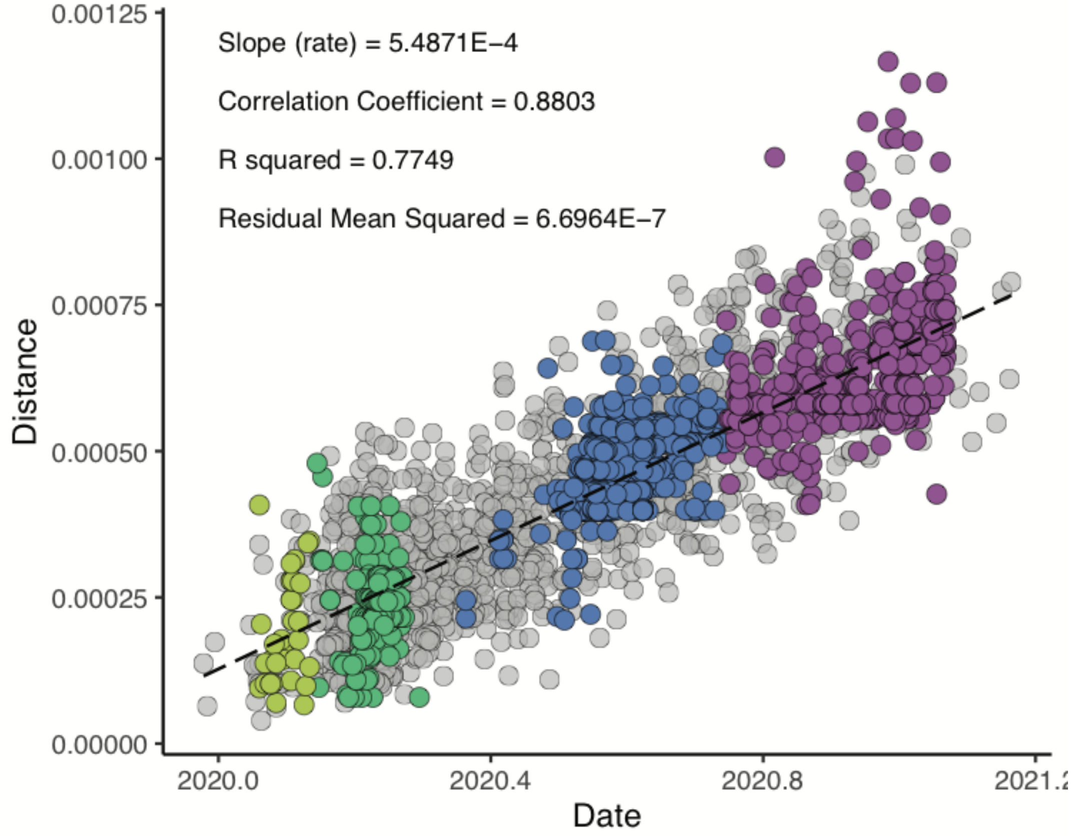


**Supplementary Fig. 2 | Root-to-tip regression analysis was performed in TempEst v.1.5.3.** Colors indicate pandemic waves in Hong Kong as in Supplementary Figure 1a.

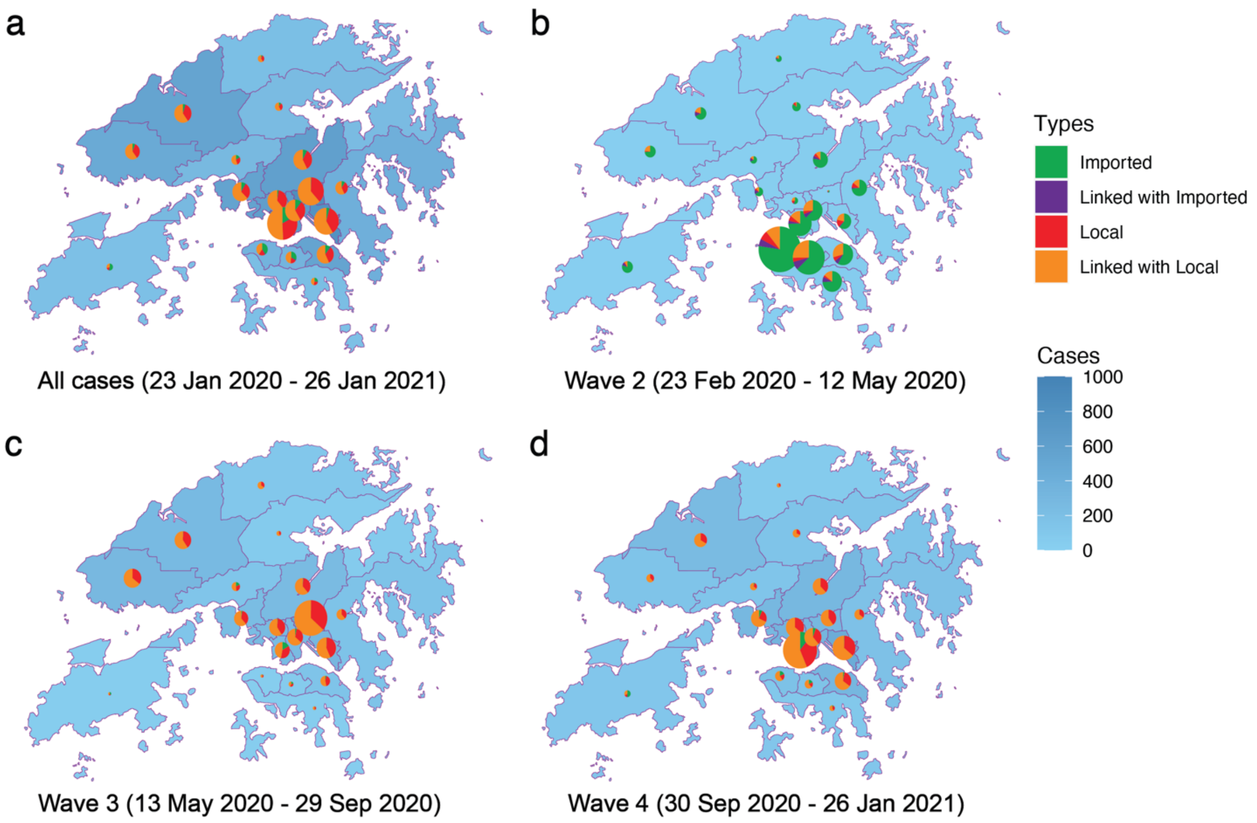


**Supplementary Fig. 3 | Geography of SARS-CoV-2 cases in Hong Kong**. (**a**) Map of Hong Kong's 18 districts shaded by the number of laboratory-confirmed cases of SARS-CoV-2. Pie charts divided by transmission types. Pie chart size reflects the number of laboratory-confirmed cases in this district (ratio of cases and radius: 1/50,000). (**b**) same as (a) based on second wave (ratio of cases and radius in pie charts: 1/5,000). (**c**) same as (a) based on third wave (ratio of cases and radius in pie charts: 1/25,000). (**d**) same as (a) based on fourth wave (ratio of cases and radius in pie charts: 1/30,000). The background of the map was obtained from GADM (version 3.6, https://gadm.org/).

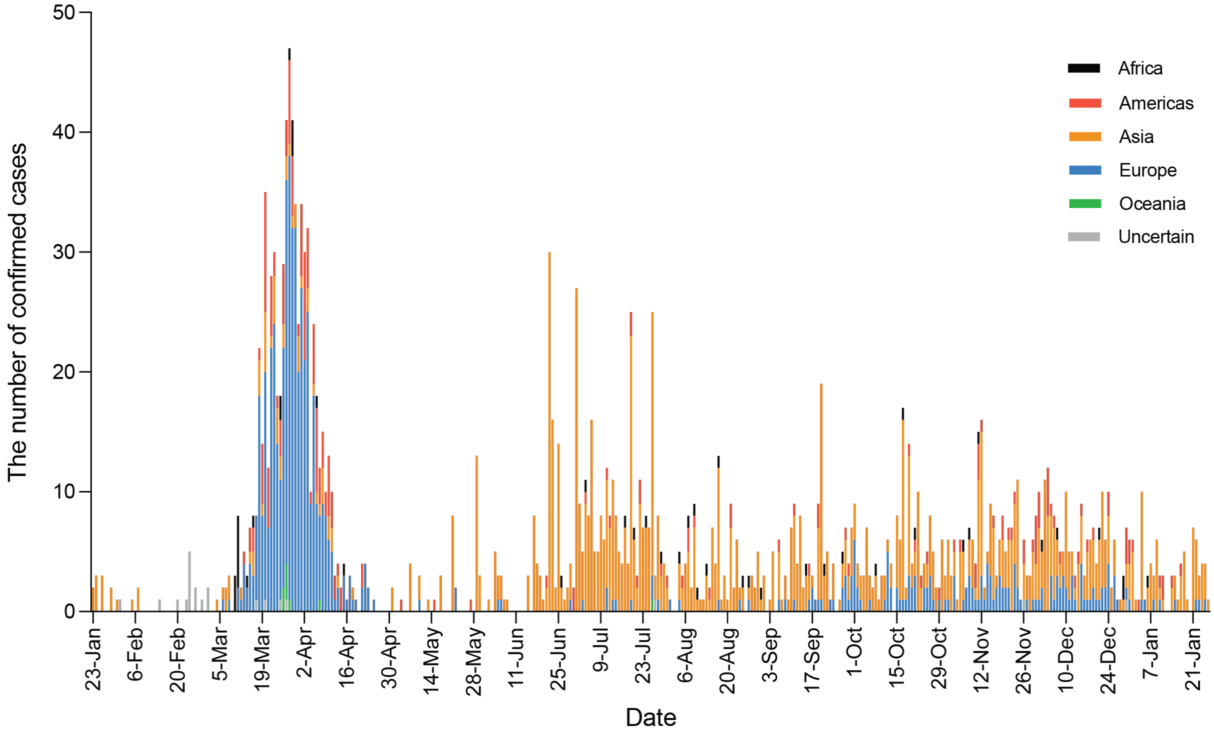


**Supplementary Fig. 4 | SARS-CoV-2 imported cases colored by continent of origin.**

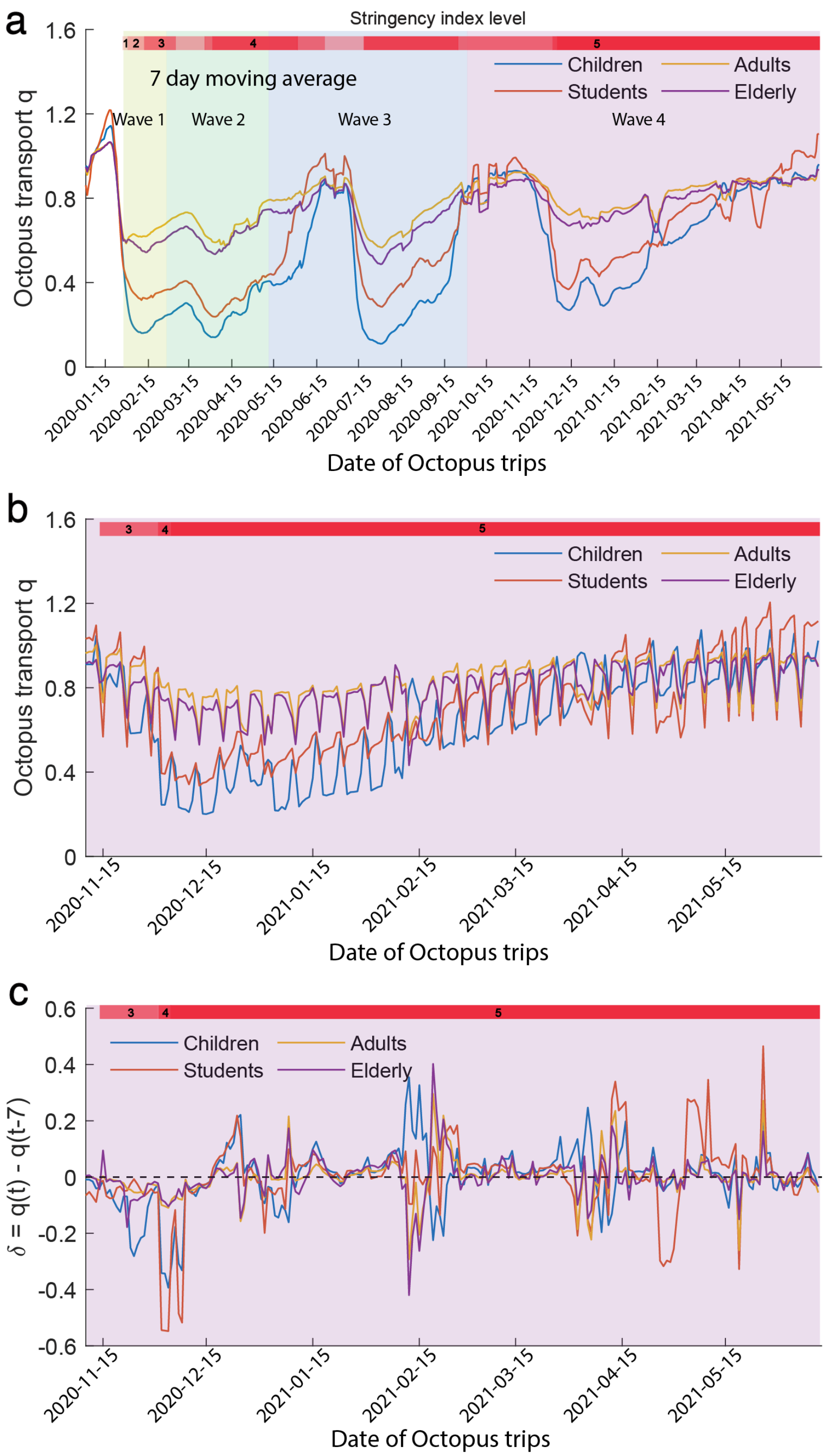


**Supplementary Fig. 5 | Octopus mobility data from January 2020 to June 2021 in Hong Kong.** Y-axes represent the normalized daily numbers of Octopus transactions according to the average number of Octopus transactions of each age group between January 1, 2020 and January 15, 2020 as benchmark (100%). Shadings indicate pandemic waves in Hong Kong as in Supplementary Figure 1a. (**a**) 7-day moving average of children, students, adults, and the elderly from January 2020 to June 2021. (**b**) daily numbers of Octopus transactions from November 2020 to June 2021. (**c**) The difference of daily numbers of Octopus transactions between seven days.

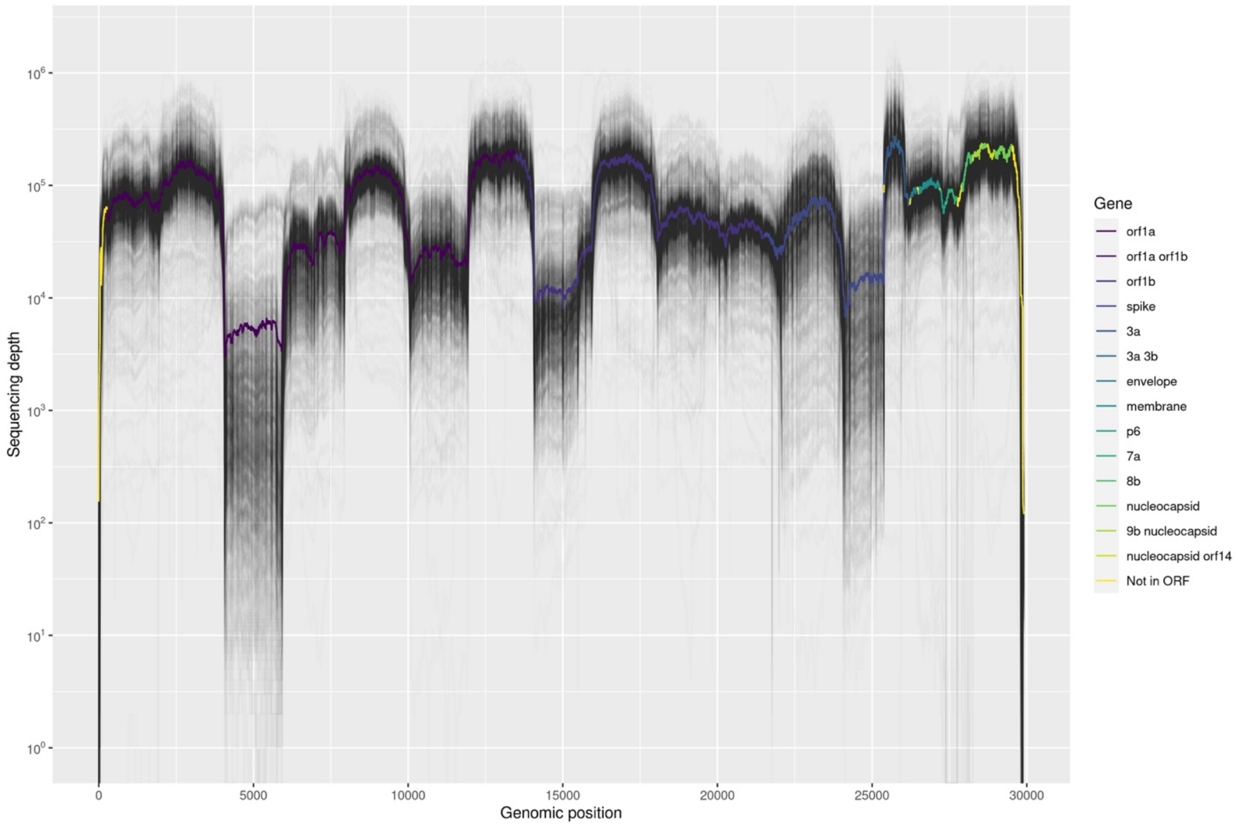


**Supplementary Fig. 6 | Sequencing depth of NGS data across the genome.** The colored line represents the average read depth at each genomic position.

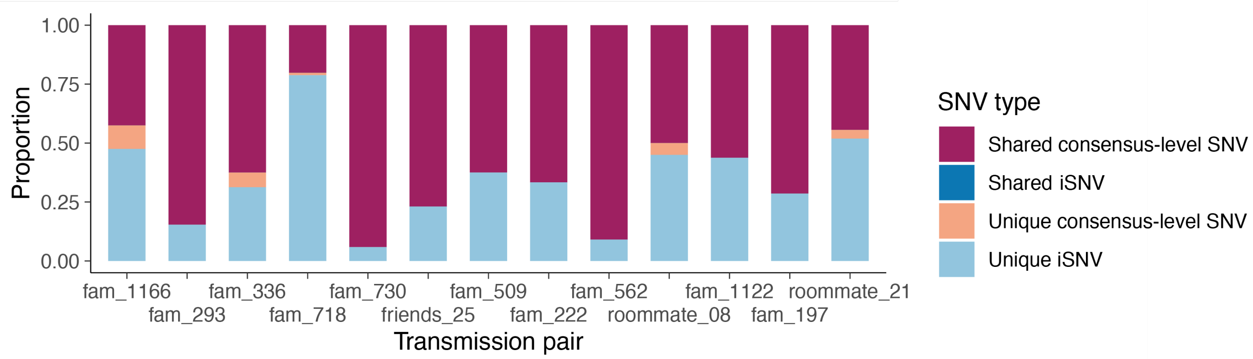


**Supplementary Fig. 7 | Proportion of shared mutations between samples from established transmission pairs.** The graph represents the SNVs profiles (variants at nucleotide level) of donor-recipient transmission pairs (In parallel to Figure 4a).

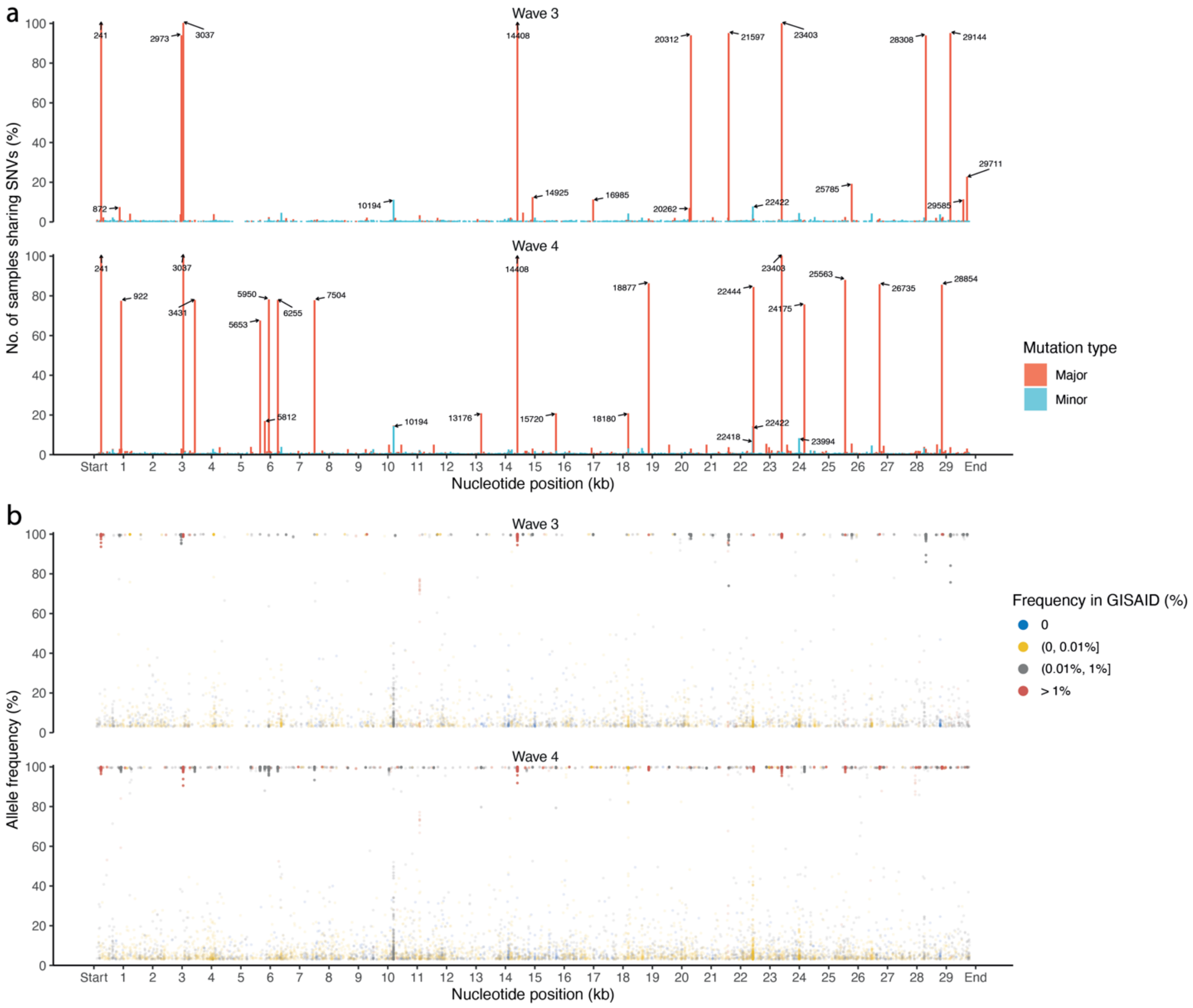


**Supplementary Data Fig. 8 | Frequencies and dynamics of SNVs in Hong Kong.** (**a**) Percentage of samples that share mutations in third and fourth wave epidemics. The color codes for consensus-level (major) and intra-host (minor) mutations at different genomic sites. High-frequency variant sites (n=36, identified in at least 5% of the respective samples) are labeled. (**b**) Relative mutation frequency (allele frequency) of SNVs. The SNVs are colored by the frequencies of mutations reported in GISIAD dataset.

## Supplementary tables

**Supplementary Table 1 | Cases and genome sequences from waves of SARS-CoV-2 in Hong Kong**

| Wave | Duration | Peak of wave (no. of cases on peak day) | Travel-related cases [b] | Community cases [c] | Total confirmed cases | Genomes sequenced | Changes in stringency [d] |
|---|---|---|---|---|---|---|---|
| 1 | 23-Jan to 22-Feb | 9-Feb (10 cases) | 17 | 53 | 70 | 36 | 1-2-3 |
| 2 | 23-Feb to 12-May | 27-Mar (65 cases) | 705 | 273 | 978 | 206 | 3-2-3-4 |
| 3 | 13-May to 29-Sep | 30-Jul (149 cases) | 647 | 3385 | 4032 | 953 | 4-3-2-4-3 |
| 4[a] | 30-Sep to 26-Jan 2021 | 29-Nov (115 cases) | 629 | 4515 | 5144 | 704 | 3-4-5 |

[a] Wave four is summarized until 26-Jan-2021 for this study. [b] Imported cases only. [c] Epidemiologically linked with imported cases, local cases, and epidemiologically linked with local cases. [d] A-B denotes the stringency index level (based on Oxford COVID-19 Government Response Tracker, see Methods) change from A to B (e.g., 1-2-3 means that the stringency index level was increased from 1 to 2 to 3).

**Supplementary Table 2 | Genome sequencing and population statistics of Hong Kong districts**

| Districts | SARS-COV-2 sample size [a] | Sequenced [b] | Latitude [c] | Longitude [c] | Population size [d] | Population density [d] (people per km$^2$) | Median monthly household income [d] (HK$) |
|---|---|---|---|---|---|---|---|
| Central and Western | 365 | 58 | 22.282150 | 114.156880 | 240,500 | 19,171 | 41,400 |
| Eastern | 548 | 81 | 22.284030 | 114.224220 | 545,600 | 30,336 | 34,300 |
| Southern | 219 | 42 | 22.246760 | 114.174134 | 264,600 | 6,812 | 32,800 |
| Wan Chai | 348 | 76 | 22.279680 | 114.171680 | 178,400 | 16,973 | 44,100 |
| Sham Shui Po | 626 | 126 | 22.330700 | 114.162163 | 416,500 | 44,517 | 24,300 |
| Kowloon City | 646 | 140 | 22.328290 | 114.191490 | 419,900 | 41,919 | 30,000 |
| Kwun Tong | 804 | 153 | 22.313260 | 114.225810 | 688,500 | 61,075 | 22,500 |
| Wong Tai Sin | 841 | 132 | 22.342140 | 114.195830 | 416,100 | 44,729 | 25,500 |
| Yau Tsim Mong | 977 | 216 | 22.321320 | 114.172580 | 329,900 | 47,177 | 30,000 |
| Islands | 203 | 38 | 22.210370 | 114.028800 | 186,500 | 1,054 | 28,400 |
| Kwai Tsing | 566 | 124 | 22.354880 | 114.084010 | 502,400 | 21,528 | 24,700 |
| North | 207 | 43 | 22.494711 | 114.138123 | 314,100 | 2,301 | 30,400 |
| Sai Kung | 400 | 78 | 22.381540 | 114.270393 | 472,500 | 3,645 | 36,500 |
| Sha Tin | 601 | 99 | 22.387159 | 114.195229 | 688,100 | 10,014 | 29,700 |
| Tai Po | 246 | 50 | 22.450840 | 114.164223 | 306,800 | 2,254 | 25,800 |
| Tsuen Wan | 288 | 66 | 22.374630 | 114.115097 | 311,800 | 5,034 | 32,600 |
| Tuen Mun | 473 | 127 | 22.396910 | 113.974411 | 495,100 | 5,964 | 25,000 |
| Yuen Long | 553 | 134 | 22.445570 | 114.022293 | 645,000 | 4,711 | 27,000 |

[a] There are 1313 cases that have unknown location information. [b] There are 116 genomes that have unknown location information. [c] Data from https://www.latlong.net/. [d] Data from the Census and Statistics Department (Hong Kong) in 2019.

**Supplementary Table 3 | Travel related cases and epidemiologically linked with imported cases within seven Hong Kong monophyletic clades (over 10 community cases)**

| Number of samples within lineage | Travel related case ID | Report date | District | Origin [a] |
|---|---|---|---|---|
| 902 | 349 | 2020-07-15 | Unknown | Philippines |
| | 1156 | 2020-09-11 | Unknown | Philippines |
| | 783 | 2020-08-02 | Eastern | Philippines |
| 552 | 1180 | 2020-09-20 | Kowloon City | Nepal |
| | 1182 | 2020-09-20 | Yau Tsim Mong | Nepal |
| | 1209 | 2020-10-05 | Yau Tsim Mong | Nepal |
| | 1183 | 2020-09-20 | Yau Tsim Mong | Nepal |
| | 1211 | 2020-10-06 | Yau Tsim Mong | Nepal |
| | 1203 | 2020-10-04 | Yau Tsim Mong | Nepal |
| | 1208 | 2020-10-05 | Yau Tsim Mong | Nepal |
| | 1184 | 2020-09-20 | Yau Tsim Mong | Nepal |
| | 1181 | 2020-09-20 | Kowloon City | Nepal |
| 92 | NA[b] | | | |
| 33 | 1596 | 2020-12-24 | Central and Western | United Kingdom |
| | 1591 | 2020-12-22 | Wan Chai | United Kingdom |
| | 1592 | 2020-12-23 | Yau Tsim Mong | India |
| | 1593 | 2020-12-23 | Yau Tsim Mong | India |
| | 1590 | 2020-12-22 | Wan Chai | India |
| | 1586 | 2020-12-21 | NA | India |
| 29 | 84 | 2020-03-21 | Yuen Long | United Kingdom |
| | 91 | 2020-03-22 | Tsuen Wan | United Kingdom |
| | 81 | 2020-03-20 | Eastern | United Kingdom |
| | 68 | 2020-03-18 | Shatin | United Kingdom |
| | 78 | 2020-03-20 | Eastern | United Kingdom |
| | 111 | 2020-03-24 | Central and Western | United Kingdom |
| | 93 | 2020-03-22 | Eastern | United Kingdom |
| | 92 | 2020-03-22 | Eastern | United Kingdom |
| | 77 | 2020-03-20 | Kwai Tsing | United Kingdom |
| | 71 | 2020-03-20 | Central and Western | United Kingdom |
| | 90 | 2020-03-22 | Tsuen Wan | United Kingdom |
| | 82 | 2020-03-20 | Shatin | United Kingdom |
| | 83 | 2020-03-21 | Yuen Long | United Kingdom |
| 19 | NA[b] | | | |
| 16 | 256 | 2020-07-01 | Shatin | United States of America |

[a] This result is based on epidemiological data. [b] No travel-related cases.

**Supplementary Table 4 | Distribution of PANGO lineages across HK-wave3 and HK-wave4A clades**

| HK clade | PANGO lineage | Number of genomes |
|---|---|---|
| HK-wave3 | B.1.1.63 | 888 |
| | B.1.1 | 5 |
| | B.1.1.220 | 6 |
| | B.1.1.192 | 2 |
| | B.1.1.398 | 1 |
| HK-wave4A | B.1.36.27 | 424 |
| | B.1.36 | 124 |
| | B.1.36.29 | 2 |
| | B.1.36.10 | 1 |
| | B.1.36.34 | 1 |

**Supplementary Table 5 | Sample numbers associated with different transmission settings in HK-wave3, HK-wave4A and all community cases in waves three and four based on epidemiological data**

| **Transmission setting** | **Wave three community cases (epidemiological data)** | **Wave four community cases (epidemiological data)** | **HK-wave3 clade (sequenced data)** | **HK-wave4A clade (sequenced data)** |
|---|---|---|---|---|
| Social | 408 | 802 | 114 | 130 |
| Rche/Rchd [a] | 145 | 117 | 47 | 4 |
| Family/Roommate | 1800 | 2352 | 470 | 226 |
| Unknown/Sporadic | 661 | 763 | 149 | 84 |
| Work | 354 | 421 | 118 | 81 |
| Nosocomial | 17 | 60 | 4 | 27 |

[a] Residential care homes for the elderly and disabled.

**Supplementary Table 6 | High-frequency SNVs identified in third and fourth waves in Hong Kong**

| Position | Wave | Gene | Synonymous mutation | Mutation at nucleotide | Mutation at amino acid | No samples with consensus-level SNVs | No samples with iSNVs | Frequency in GISAID | Proportion in GISAID (%) |
|---|---|---|---|---|---|---|---|---|---|
| 241 | Wave 3 & 4 | Not in ORF | Not in ORF | C241T | NA | 1598 | 0 | 377531 | 94.59 |
| 3037 | Wave 3 & 4 | nsp3 | TRUE | C3037T | F106F | 1600 | 0 | 381081 | 95.48 |
| 10194 | Wave 3 & 4 | nsp5 | FALSE | A10194T\|G | E47V\|G | 2 | 392 | 43 | 0.01 |
| 14408 | Wave 3 & 4 | nsp12_2 | FALSE | C14408T | P314L | 1595 | 0 | 380926 | 95.44 |
| 22422 | Wave 3 & 4 | S | FALSE | A22422G\|T | D287G\|V | 5 | 322 | 28 | 0.01 |
| 23403 | Wave 3 & 4 | S | FALSE | A23403G | D614G | 1597 | 0 | 381443 | 95.57 |
| 872 | Wave 3 | nsp2 | FALSE | G872A\|T\|C | D23N\|Y\|H | 82 | 4 | 679 | 0.17 |
| 2973 | Wave 3 | nsp3 | FALSE | C2973T | A85V | 859 | 1 | 1500 | 0.38 |
| 14925 | Wave 3 | nsp12_2 | TRUE | C14925T | V486V | 128 | 0 | 849 | 0.21 |
| 16985 | Wave 3 | nsp13 | FALSE | C16985T | T250I | 98 | 1 | 141 | 0.04 |
| 20262 | Wave 3 | nsp15 | TRUE | A20262G | L214L | 60 | 1 | 522 | 0.13 |
| 20312 | Wave 3 | nsp15 | FALSE | C20312T | A231V | 859 | 0 | 148 | 0.04 |
| 21597 | Wave 3 | S | FALSE | C21597T | S12F | 874 | 1 | 633 | 0.16 |
| 25785 | Wave 3 | ORF3a | FALSE | G25785T\|A | W131C\|* | 202 | 8 | 1864 | 0.47 |
| 28308 | Wave 3 | N | FALSE | C28308G\|T | A12G\|V | 857 | 1 | 160 | 0.04 |
| 29144 | Wave 3 | N | TRUE | C29144T | L291L | 874 | 0 | 606 | 0.15 |
| 29585 | Wave 3 | ORF10 | FALSE | C29585T | P10S | 97 | 2 | 701 | 0.18 |
| 29711 | Wave 3 | Not in ORF | Not in ORF | G29711T | NA | 220 | 0 | 596 | 0.15 |
| 922 | Wave 4 | nsp2 | TRUE | G922A | L39L | 549 | 0 | 223 | 0.06 |
| 3431 | Wave 4 | nsp3 | FALSE | G3431T | V238L | 552 | 0 | 426 | 0.11 |
| 5653 | Wave 4 | nsp3 | TRUE | T5653C | Y978Y | 479 | 0 | 109 | 0.03 |
| 5812 | Wave 4 | nsp3 | TRUE | C5812T | D1031D | 117 | 1 | 903 | 0.23 |
| 5950 | Wave 4 | nsp3 | FALSE\|TRUE | G5950T\|A | K1077N\|K | 1110 | 12 | 1175 | 0.29 |
| 6255 | Wave 4 | nsp3 | FALSE | C6255T | A1179V | 552 | 0 | 543 | 0.14 |
| 7504 | Wave 4 | nsp3 | TRUE | C7504T | Y1595Y | 551 | 0 | 170 | 0.04 |
| 13176 | Wave 4 | nsp10 | FALSE | C13176T | T51I | 144 | 0 | 235 | 0.06 |
| 15720 | Wave 4 | nsp12_2 | TRUE | C15720T | D751D | 144 | 3 | 1377 | 0.35 |
| 18180 | Wave 4 | nsp14 | TRUE | G18180A | K47K | 144 | 0 | 33 | 0.01 |
| 18877 | Wave 4 | nsp14 | TRUE | C18877T | L280L | 617 | 1 | 23681 | 5.93 |
| 22418 | Wave 4 | S | FALSE | A22418G | T286A | 1 | 76 | 1 | 0 |
| 22444 | Wave 4 | S | TRUE | C22444T | D294D | 603 | 1 | 7893 | 1.98 |
| 23994 | Wave 4 | S | FALSE | A23994G | K811R | 1 | 93 | 9 | 0 |
| 24175 | Wave 4 | S | TRUE | T24175C | A871A | 536 | 0 | 59 | 0.01 |
| 25563 | Wave 4 | ORF3a | FALSE | G25563C\|T | Q57H | 631 | 10 | 89470 | 22.42 |
| 26735 | Wave 4 | M | TRUE | C26735T | Y71Y | 614 | 1 | 21029 | 5.27 |

| | | | | | | | | | |
|---|---|---|---|---|---|---|---|---|---|
| 28854 | Wave 4 | N | FALSE | C28854T | S194L | 611 | 3 | 23575 | 5.91 |

**Supplementary Table 7 | iSNVs identified in Hong Kong cases (frequency in GISAID ≥1%)**

| Position | Gene | Frequency in HK | (%) | Silent mutation | Frequency in GISAID | Frequency in GISAID (%) | Mutation (nucleotide) | Mutation (amino acid) |
|---|---|---|---|---|---|---|---|---|
| 28883 | N | 25 | 1.56 | FALSE | 152044 | 38.09 | G28883C | G610R |
| 26801 | M | 10 | 0.62 | TRUE | 86175 | 21.59 | C26801G\|T | L279L |
| 22227 | S | 11 | 0.69 | FALSE | 85789 | 21.49 | C22227T | A665V |
| 21255 | nsp16 | 9 | 0.56 | TRUE | 85524 | 21.43 | G21255T\|C | A597A |
| 6286 | nsp3 | 12 | 0.75 | TRUE | 85407 | 21.4 | C6286T | T3567T |
| 29645 | ORF10 | 10 | 0.62 | FALSE | 85145 | 21.33 | G29645T | V88L |
| 28932 | N | 9 | 0.56 | FALSE | 85057 | 21.31 | C28932A\|T | A659D\|V |
| 445 | nsp1 | 10 | 0.62 | TRUE | 84963 | 21.29 | T445C | V180V |
| 1059 | nsp2 | 13 | 0.81 | FALSE | 59725 | 14.96 | C1059T | T254I |
| 27944 | ORF8 | 7 | 0.44 | TRUE | 57276 | 14.35 | C27944T | H51H |
| 23604 | S | 15 | 0.94 | FALSE | 56514 | 14.16 | C23604T\|G\|A | P2042L\|R\|H |
| 23063 | S | 16 | 1 | FALSE | 53756 | 13.47 | A23063T\|G | N1501Y\|D |
| 5986 | nsp3 | 13 | 0.81 | TRUE | 53506 | 13.41 | C5986T | F3267F |
| 28977 | N | 16 | 1 | FALSE | 53437 | 13.39 | C28977T | S704F |
| 3267 | nsp3 | 18 | 1.12 | FALSE | 52970 | 13.27 | C3267T | T548I |
| 14676 | nsp12_2 | 12 | 0.75 | TRUE | 52930 | 13.26 | C14676T | P1209P |
| 23709 | S | 11 | 0.69 | FALSE | 52646 | 13.19 | C23709T | T2147I |
| 27972 | ORF8 | 12 | 0.75 | FALSE | 52586 | 13.18 | C27972T | Q79* |
| 24914 | S | 12 | 0.75 | FALSE | 52551 | 13.17 | G24914T\|C | D3352Y\|H |
| 15279 | nsp12_2 | 12 | 0.75 | TRUE | 52500 | 13.15 | C15279T | H1812H |
| 23271 | S | 11 | 0.69 | FALSE | 52472 | 13.15 | C23271A | A1709D |
| 28048 | ORF8 | 12 | 0.75 | FALSE | 52430 | 13.14 | G28048A\|T | R155K\|I |
| 24506 | S | 11 | 0.69 | FALSE | 52370 | 13.12 | T24506G | S2944A |
| 16176 | nsp12_2 | 12 | 0.75 | TRUE | 52366 | 13.12 | T16176C | T2709T |
| 28111 | ORF8 | 10 | 0.62 | FALSE | 52347 | 13.12 | A28111G | Y218C |
| 6954 | nsp3 | 11 | 0.69 | FALSE | 52344 | 13.11 | T6954C | I4235T |
| 5388 | nsp3 | 7 | 0.44 | FALSE | 52288 | 13.1 | C5388A | A2669D |
| 913 | nsp2 | 14 | 0.87 | TRUE | 52196 | 13.08 | C913T | S108S |
| 204 | Not in ORF | 5 | 0.31 | Not in ORF | 47971 | 12.02 | G204T\|A | NA |
| 21614 | S | 4 | 0.25 | FALSE | 39312 | 9.85 | C21614T | L52F |
| 20268 | nsp15 | 7 | 0.44 | TRUE | 26684 | 6.69 | A20268G | L648L |
| 27964 | ORF8 | 6 | 0.37 | FALSE | 24942 | 6.25 | C27964T | S71L |
| 28869 | N | 7 | 0.44 | FALSE | 23983 | 6.01 | C28869T | P596L |
| 313 | nsp1 | 24 | 1.5 | TRUE | 21829 | 5.47 | C313T | L48L |
| 22992 | S | 9 | 0.56 | FALSE | 21826 | 5.47 | G22992C\|A\|T | S1430T\|N\|I |

| | | | | | | | | |
|---|---|---|---|---|---|---|---|---|
| 10319 | nsp5 | 6 | 0.37 | FALSE | 20462 | 5.13 | C10319T\|A | L265F\|I |
| 11083 | nsp6 | 38 | 2.37 | FALSE\|TRUE | 20168 | 5.05 | G11083T\|A | L111F\|L |
| 17615 | nsp13 | 9 | 0.56 | FALSE | 19741 | 4.95 | A17615G | K1379R |
| 28975 | N | 5 | 0.31 | FALSE | 19390 | 4.86 | G28975C\|T\|A | M702I |
| 21304 | nsp16 | 6 | 0.37 | FALSE | 18702 | 4.69 | C21304T | R646C |
| 18424 | nsp14 | 4 | 0.25 | FALSE | 17916 | 4.49 | A18424G | N385D |
| 25907 | ORF3a | 4 | 0.25 | FALSE | 17673 | 4.43 | G25907T | G515V |
| 28472 | N | 4 | 0.25 | FALSE | 17473 | 4.38 | C28472T | P199S |
| 14805 | nsp12_2 | 4 | 0.25 | TRUE | 14592 | 3.66 | C14805T | Y1338Y |
| 4543 | nsp3 | 5 | 0.31 | TRUE | 11630 | 2.91 | C4543T | T1824T |
| 25710 | ORF3a | 3 | 0.19 | TRUE | 11496 | 2.88 | C25710T | L318L |
| 15766 | nsp12_2 | 2 | 0.12 | FALSE | 11473 | 2.87 | G15766T | V2299L |
| 17019 | nsp13 | 5 | 0.31 | FALSE\|TRUE | 11208 | 2.81 | G17019T\|A | E783D\|E |
| 9526 | nsp4 | 4 | 0.25 | FALSE | 11146 | 2.79 | G9526T\|C | M972I |
| 13993 | nsp12_2 | 2 | 0.12 | FALSE | 11064 | 2.77 | G13993T | A526S |
| 11497 | nsp6 | 2 | 0.12 | TRUE | 11042 | 2.77 | C11497T | Y525Y |
| 26876 | M | 2 | 0.12 | TRUE | 10983 | 2.75 | T26876C | I354I |
| 16889 | nsp13 | 2 | 0.12 | FALSE | 10934 | 2.74 | A16889G | K653R |
| 29399 | N | 2 | 0.12 | FALSE | 10916 | 2.73 | G29399A | A1126T |
| 23401 | S | 3 | 0.19 | FALSE | 10620 | 2.66 | G23401T | Q1839H |
| 5629 | nsp3 | 2 | 0.12 | TRUE | 10398 | 2.61 | G5629T | T2910T |
| 15324 | nsp12_2 | 4 | 0.25 | TRUE | 10168 | 2.55 | C15324T | N1857N |
| 29734 | Not in ORF | 5 | 0.31 | Not in ORF | 9939 | 2.49 | G29734T\|C | NA |
| 222 | Not in ORF | 2 | 0.12 | Not in ORF | 9834 | 2.46 | C222T | NA |
| 17104 | nsp13 | 5 | 0.31 | FALSE | 8996 | 2.25 | C17104T | H868Y |
| 22879 | S | 3 | 0.19 | FALSE | 8965 | 2.25 | C22879A | N1317K |
| 29366 | N | 3 | 0.19 | FALSE | 8923 | 2.24 | C29366T | P1093S |
| 7767 | nsp3 | 12 | 0.75 | FALSE | 8618 | 2.16 | T7767C | I5048T |
| 8047 | nsp3 | 3 | 0.19 | TRUE | 8583 | 2.15 | C8047T | Y5328Y |
| 8083 | nsp3 | 5 | 0.31 | FALSE | 8156 | 2.04 | G8083A | M5364I |
| 20661 | nsp16 | 3 | 0.19 | TRUE | 8129 | 2.04 | T20661C | S3S |
| 29402 | N | 10 | 0.62 | FALSE | 7511 | 1.88 | G29402T\|C | D1129Y\|H |
| 27800 | ORF7b | 3 | 0.19 | TRUE | 7440 | 1.86 | C27800A | A39A |
| 28725 | N | 3 | 0.19 | FALSE | 7311 | 1.83 | C28725T | P452L |
| 9286 | nsp4 | 17 | 1.06 | TRUE | 7271 | 1.82 | C9286T | N732N |
| 10097 | nsp5 | 6 | 0.37 | FALSE | 6822 | 1.71 | G10097A\|T\|C | G43S\|C\|R |
| 18028 | nsp13 | 6 | 0.37 | FALSE | 6674 | 1.67 | G18028T | A1792S |
| 21855 | S | 5 | 0.31 | FALSE | 6653 | 1.67 | C21855T | S293F |
| 21575 | S | 25 | 1.56 | FALSE | 6592 | 1.65 | C21575T | L13F |
| 12988 | nsp9 | 5 | 0.31 | FALSE | 6563 | 1.64 | G12988T\|C | M303I |

| | | | | | | | | |
|---|---|---|---|---|---|---|---|---|
| 26972 | M | 3 | 0.19 | TRUE | 6559 | 1.64 | T26972C | R450R |
| 15598 | nsp12_2 | 3 | 0.19 | FALSE | 6554 | 1.64 | G15598A | V2131I |
| 24910 | S | 5 | 0.31 | TRUE | 6544 | 1.64 | T24910C\|G | T3348T |
| 2453 | nsp2 | 4 | 0.25 | FALSE | 6270 | 1.57 | C2453T | L1648F |
| 28651 | N | 4 | 0.25 | TRUE | 6143 | 1.54 | C28651T | N378N |
| 28887 | N | 8 | 0.5 | FALSE | 6124 | 1.53 | C28887T | T614I |
| 19839 | nsp15 | 2 | 0.12 | TRUE | 6093 | 1.53 | T19839C | N219N |
| 23731 | S | 4 | 0.25 | TRUE | 6068 | 1.52 | C23731T | T2169T |
| 10323 | nsp5 | 11 | 0.69 | FALSE | 5911 | 1.48 | A10323G | K269R |
| 11396 | nsp6 | 3 | 0.19 | FALSE | 5509 | 1.38 | C11396T | L424F |
| 2416 | nsp2 | 6 | 0.37 | TRUE | 5362 | 1.34 | C2416T | Y1611Y |
| 10870 | nsp5 | 5 | 0.31 | TRUE | 5156 | 1.29 | G10870T\|A | L816L |
| 9745 | nsp4 | 3 | 0.19 | TRUE | 5069 | 1.27 | C9745T | Y1191Y |
| 20451 | nsp15 | 4 | 0.25 | TRUE | 4978 | 1.25 | C20451T | N831N |
| 22346 | S | 4 | 0.25 | FALSE | 4665 | 1.17 | G22346T | A784S |
| 28087 | ORF8 | 2 | 0.12 | FALSE | 4662 | 1.17 | C28087T | A194V |
| 26424 | E | 7 | 0.44 | TRUE | 4656 | 1.17 | T26424C | S180S |
| 8603 | nsp4 | 3 | 0.19 | FALSE | 4626 | 1.16 | T8603C | F49L |
| 13536 | nsp12_2 | 3 | 0.19 | TRUE | 4547 | 1.14 | C13536T | Y69Y |
| 15480 | nsp12_2 | 3 | 0.19 | TRUE | 4530 | 1.13 | C15480A\|T | T2013T |
| 3177 | nsp3 | 3 | 0.19 | FALSE | 4398 | 1.1 | C3177T | P458L |
| 8917 | nsp4 | 8 | 0.5 | TRUE | 4375 | 1.1 | C8917T | F363F |
| 4002 | nsp3 | 4 | 0.25 | FALSE | 4288 | 1.07 | C4002T | T1283I |
| 19524 | nsp14 | 3 | 0.19 | TRUE | 4175 | 1.05 | C19524T | L1485L |
| 29179 | N | 5 | 0.31 | TRUE | 4162 | 1.04 | G29179T\|A\|C | P906P |
| 25437 | ORF3a | 4 | 0.25 | FALSE | 4088 | 1.02 | G25437T | L45F |
| 22388 | S | 2 | 0.12 | TRUE | 4038 | 1.01 | C22388T | L826L |
| 28253 | ORF8 | 43 | 2.69 | TRUE | 3982 | 1 | C28253T | F360F |

**Supplementary Table 8 | Estimation on bottleneck size of transmission pairs**

| Transmission pair | Variant calling threshold | Donor | Recipient | Bottleneck size | CI lower | CI upper |
|---|---|---|---|---|---|---|
| Cluster_fam_1122 | 0.03 | 8773 | 8772 | 3 | 1 | 10 |
| Cluster_fam_1166 | 0.03 | 9042 | 9041 | 2 | 1 | 4 |
| Cluster_fam_197 | 0.03 | 1905 | 2168 | 1 | 0 | 9 |
| Cluster_fam_222 | 0.03 | 2172 | 2317 | 1 | 0 | 27 |
| Cluster_fam_293 | 0.03 | 2735 | 2609 | 1 | 0 | 202 |
| Cluster_fam_336 | 0.03 | 2989 | 2962 | 1 | 0 | 13 |
| Cluster_fam_509 | 0.03 | 3970 | 3612 | 1 | 0 | 22 |
| Cluster_fam_562 | 0.03 | 4306 | 4307 | NA | NA | NA |
| Cluster_fam_718 | 0.03 | 5399 | 5444 | 1 | 0 | 13 |
| Cluster_fam_730 | 0.03 | 5539 | 5577 | NA | NA | NA |
| Cluster_friends_25 | 0.03 | 1839 | 2047 | 1 | 0 | 10 |
| Cluster_roommate_08 | 0.03 | 2721 | 2545 | 1 | 0 | 5 |
| Cluster_roommate_21 | 0.03 | 4075 | 4208 | 1 | 0 | 4 |

**Supplementary Table 9 | Highly shared variant sites (allele frequency ≥3% and were found in >1% of the HK samples) located within or related to PCR primer binding regions**

| Position | Number of samples with SNV | Proportion in HK samples |
|---|---|---|
| 1912 | 18 | 0.01124297 |
| 1947 | 551 | 0.3441599 |
| 15487 | 28 | 0.01748907 |
| 15489 | 1105 | 0.69019363 |
| 15494 | 1151 | 0.71892567 |
| 18100 | 347 | 0.21673954 |
| 24082 | 178 | 0.11118051 |
| 24091 | 860 | 0.53716427 |
| 26060 | 550 | 0.34353529 |
| 29799 | 21 | 0.0131168 |

**Supplementary Table 10 | Gene annotation of SARS-CoV-2 Genome (nucleotide positions base on reference sequence Wuhan-Hu-1, GenBank: MN908947.3)**

| Gene segment | Start | Stop |
|---|---|---|
| nsp1 | 266 | 805 |
| nsp2 | 806 | 2719 |
| nsp3 | 2720 | 8554 |
| nsp4 | 8555 | 10054 |
| nsp5 | 10055 | 10972 |
| nsp6 | 10973 | 11842 |
| nsp7 | 11843 | 12091 |
| nsp8 | 12092 | 12685 |
| nsp9 | 12686 | 13024 |
| nsp10 | 13025 | 13441 |
| nsp12_1 | 13442 | 13468 |
| nsp12_2 | 13468 | 16236 |
| nsp13 | 16237 | 18039 |
| nsp14 | 18040 | 19620 |
| nsp15 | 19621 | 20658 |
| nsp16 | 20659 | 21555 |
| S | 21563 | 25384 |
| ORF3a | 25393 | 26220 |
| E | 26245 | 26472 |
| M | 26523 | 27191 |
| ORF6 | 27202 | 27387 |
| ORF7a | 27394 | 27753 |
| ORF7b | 27762 | 27887 |
| ORF8 | 27894 | 28259 |
| N | 28274 | 29533 |
| ORF10 | 29558 | 29674 |
