# Supplementary material for: Genomic epidemiology of SARS-CoV-2 under an elimination strategy in Hong Kong
Source: Nat Commun. 2022 Feb 8;13:736. doi: 10.1038/s41467-022-28420-7 (PMC8825829; doi:10.1038/s41467-022-28420-7)
Supplement: Supplementary file 3 — Description of Additional Supplementary Files [file 41467_2022_28420_MOESM3_ESM.pdf]

Description of Additional Supplementary Files

File name: Supplementary Data 1

Description: Origins of imported cases in Hong Kong.

File name: Supplementary Data 2

Description: Sample list with waves, NextClade and PANGO lineage designations.

File name: Supplementary Data 3

Description: Summary of Hong Kong monophyletic clades.

File name: Supplementary Data 4

Description: Acknowledgements to sequences obtained from GISAID (accessed on 11-June-2021).
